# Dual Knockdown of Musashi RNA-Binding Proteins MSI-1 and MSI-2 Attenuates Putative Cancer Stem Cell Characteristics and Therapy Resistance in Ovarian Cancer Cells

**DOI:** 10.3390/ijms222111502

**Published:** 2021-10-25

**Authors:** Maria T. Löblein, Isabel Falke, Hans Theodor Eich, Burkhard Greve, Martin Götte, Fabian M. Troschel

**Affiliations:** 1 Department of Radiation Oncology, University Hospital Münster, 48149 Münster, Germany; m.loeblein@uni-muenster.de (M.T.L.); isabel.falke@uni-muenster.de (I.F.); hans.eich@ukmuenster.de (H.T.E.); burkhard.greve@ukmuenster.de (B.G.); 2 Department of Gynecology and Obstetrics, University Hospital Münster, 48149 Münster, Germany; martin.goette@ukmuenster.de

**Keywords:** ovarian cancer, therapy resistance, cancer stem cell, Musashi, radioresistance, notch, cell cycle, proliferation

## Abstract

In ovarian cancer, therapy resistance mechanisms complicate cancer cell eradication. Targeting Musashi RNA-binding proteins (MSI) may increase therapeutic efficacy. Database analyses were performed to identify gene expression associations between MSI proteins and key therapy resistance and cancer stem cell (CSC) genes. Then, ovarian cancer cells were subjected to siRNA-based dual knockdown of MSI-1 and MSI-2. CSC and cell cycle gene expression was investigated using quantitative polymerase chain reaction (qPCR), western blots, and flow cytometry. Metabolic activity and chemoresistance were assessed by MTT assay. Clonogenic assays were used to quantify cell survival post-irradiation. Database analyses demonstrated positive associations between MSI proteins and putative CSC markers NOTCH, MYC, and ALDH4A1 and negative associations with NOTCH inhibitor NUMB. MSI-2 expression was negatively associated with the apoptosis regulator p21. MSI-1 and MSI-2 were positively correlated, informing subsequent dual knockdown experiments. After MSI silencing, CSC genes were downregulated, while cell cycle progression was reduced. Metabolic activity was decreased in some cancer cells. Both chemo- and radioresistance were reduced after dual knockdown, suggesting therapeutic potential. Dual knockdown of MSI proteins is a promising venue to impede tumor growth and sensitize ovarian cancer cells to irradiation and chemotherapy.

## 1. Introduction

Proteins of the Musashi family can be found in normal and tumor cells. As stem cell markers, they regulate numerous targets by binding messenger ribonucleic acids (mRNAs) via specific RNA-binding motifs. Downstream effects on cell fate include changes in differentiation, survival, proliferation, and therapy resistance [1].

Musashi consists of two variants, Musashi-1 (MSI-1) and Musashi-2 (MSI-2). Both proteins have been shown to be about 90% homologous and are also closely related in their RNA-binding specificity [2,3]. They are known to regulate the stem cell character of both somatic and germ cells [4] and are often aberrantly expressed in cancer cells [5]. Dysregulation of either or both of these highly conserved proteins can lead to cellular dysfunction, including instability and tumorigenesis in a wide array of different cancer entities [6,7]. Subsequently, expression of both Musashi proteins has been shown to be negatively associated with overall survival in cancer patients [8,9]. Recent data of our group also suggest a radioprotective role of Musashi in triple negative breast cancer as siRNA-based knockdown radiosensitized cells [10]. These effects were mainly caused by modulation of notch signaling pathway [11], cell cycle signaling [12], and DNA damage repair [13].

Ovarian cancer remains one of the most fatal diseases among women with limited therapeutic options and high rates of therapy resistance [14]. MSI-1 is known to be highly expressed in this entity and has been linked to overall survival [15]. Separate studies have shown that targeting either MSI-1 or MSI-2 may reverse paclitaxel chemoresistance [16,17]. Despite the increasing use of radiotherapy in ovarian cancer patients [18,19,20,21], MSI-related effects remain unknown.

In the present study, we aimed to investigate the therapeutic potential of targeting both MSI-1 and MSI-2 as a dual knockdown. Clinically, investigations have detailed the difficulty of targeting and inhibiting MSI-1 and MSI-2 separately given their close similarity [22]. Additionally, both proteins have been described to possess overlapping functions, making a dual inhibition attractive. Finally, MSI-1 and MSI-2 have been shown to be relevantly correlated, underlining their close relationship.

The purpose of our study was two-fold: first, we aimed to establish a relationship between the Musashi family and putative cancer stem cells (CSC) in ovarian cancer. Subsequently, second, we set out to understand the therapeutic potential of a dual Musashi knockdown focusing on proliferation as well as radio- and chemoresistance.

## 2. Results

In the present study, we aimed to establish the relationship between the Musashi family and putative cancer stem cells to understand the therapeutic potential of a dual Musashi knockdown in ovarian cancer.

### 2.1. MSI-1 and -2 Expression Correlate with Each Other and with Cancer Stem Cell-Associated Genes

**Figure 1:**
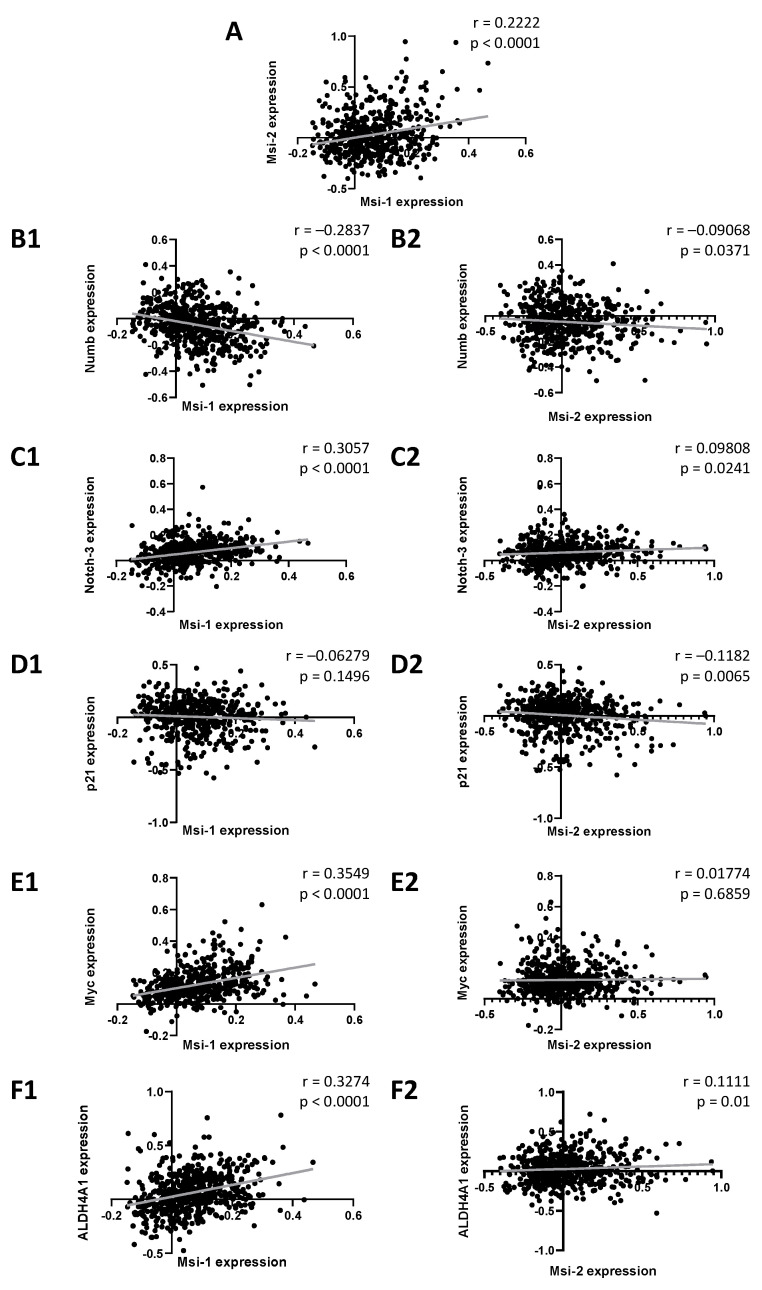
Associations between Musashi-1 and Musashi-2 expression and expression of genes relevant to cancer stem cells, cell cycle, and therapy resistance. Data are based on the GSE74357 dataset, and normalized mRNA expression is presented. Spearman correlation analyses were performed to obtain strength (r) and significance (*p*) for each test correlation.

First, we performed database analyses in 529 ovarian cancer samples to detect genes correlated with MSI-1 and -2 gene expression. When comparing MSI-1 and MSI-2 with each other (Figure 1A), a significant positive correlation was seen. Further significant correlations were identified for both MSI-1 and MSI-2 with cancer stem cell-associated genes of the notch signaling pathway: both were negatively correlated with NUMB (Figure 1(B1,B2)) and positively correlated with NOTCH-3 (Figure 1(C1,C2)). When investigating cell cycle regulators, MSI-1 was found to be positively correlated with MYC (Figure 1(E1,E2)), and MSI-2 was negatively correlated with p21 (Figure 1(D1,D2)). Additionally, significant positive correlations of ALDH4A1 with both MSI proteins were found. Here, correlation with MSI-1 (Figure 1(F1)) was stronger than correlation with MSI-2 (Figure 1(F2)).

### 2.2. MSI-1 and -2 Affect Cancer Stem Cell Characteristics

MSI is known to affect stem cell characteristics of tumor cells [5]. Since CSCs are important for maintenance of therapy resistance, we looked for alterations in expression of CSC-related genes, trying to identify involved signaling pathways.

**Figure 2:**
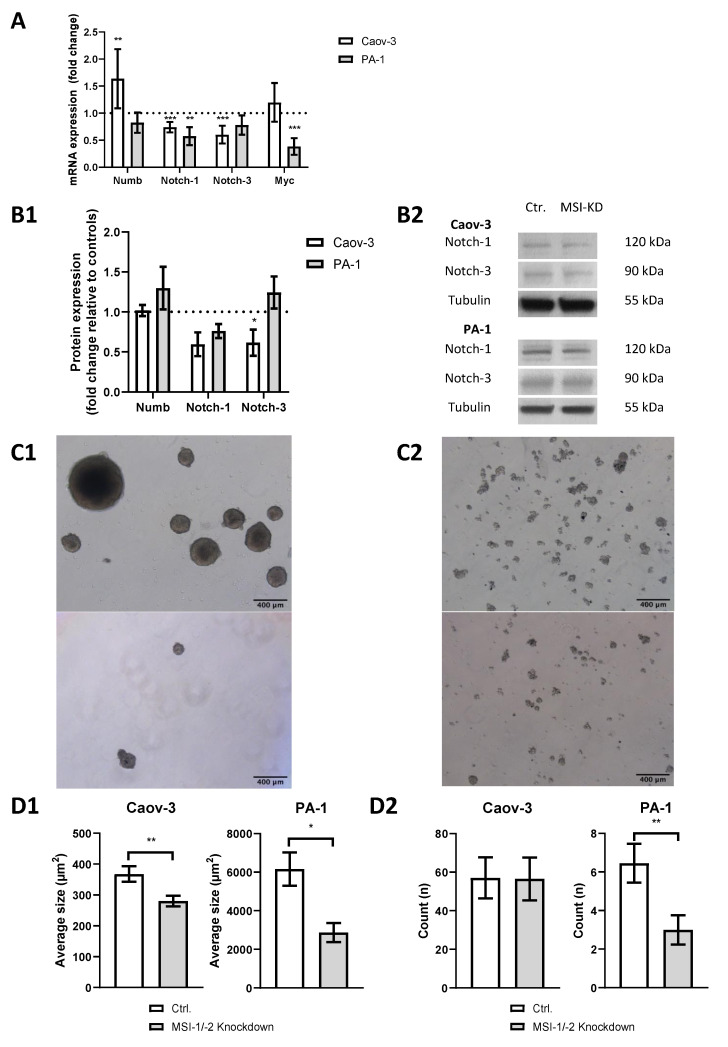
Dual inhibition of Musashi-1 and Musashi-2 impedes cancer stem cell characteristics such as notch pathway gene expression, including pathway elements NOTCH-1 and NOTCH-3 and sphere formation ability. (**A**): Quantitative polymerase chain reaction (qPCR) results. (**B1**): Western blot results. (**B2**): Representative western blot staining. All experiments were repeated at least three times in duplicates. (**C1**): Representative pictures of sphere formation in PA-1 cells (upper picture: control; lower picture: MSI dual knockdown). (**C2**): Representative pictures of sphere formation in Caov-3 cells (upper picture: control; lower picture: MSI dual knockdown). (**D1**): The average size of spheres decreases after MSI dual knockdown in Caov-3 and PA-1 cells. (**D2**): MSI double knockdown leads to a lower count of cell structures in PA-1 cells per microscopic field of view. *p* values < 0.05 were deemed significant (* *p* < 0.05; ** *p* < 0.01; *** *p* < 0.001, error bars indicate standard error of the mean (s.e.m.)).

We performed siRNA-based dual MSI knockdown and verified its success via qPCR. Both MSI-1 and MSI-2 were suppressed by more than 70% (*p* < 0.001, Supplementary Figure S1) in both cell lines. qPCR results were confirmed by Western Blot analysis (Supplementary Figure S2). In PA-1 cells, MSI-1 and MSI-2 were reduced by 90% (*p* = 0.003) and 50% (*p* = 0.05), respectively. In Caov-3 cells, MSI-1 was decreased by 45% (*p* = 0.04), while MSI-2 was only slightly decreased, barely outside the level of significance (*p* = 0.07). As for all western blot experiments, we also quantified relative protein expression levels compared to tubulin (Supplementary Figures S3 and S4). qPCR also demonstrated altered expression of several genes of the notch signaling pathway after MSI knockdown (Figure 2A): NUMB was significantly upregulated in Caov-3 cells (*p* = 0.004), while no effect was seen in PA-1 cells. However, in both cell lines, notch pathway components were down, NOTCH-1 and NOTCH-3 in Caov-3 (*p* < 0.001 and *p* < 0.001) and NOTCH-1 in PA-1 (*p* = 0.005).

Given that MSI proteins are a posttranscriptional regulator of gene expression [1,2], qPCR results were validated by Western blot analysis (Figure 2(B1,B2)). A downregulation trend of NOTCH-1 was found for both Caov-3 (*p* = 0.07) and PA-1 (*p* = 0.07) cells. The Caov-3 cell line showed a NOTCH-3 downregulation of at least 40% (*p* = 0.008), while no significant changes were seen in PA-1.

To further assess stemness properties in both cell lines, a sphere formation assay was conducted. Baseline sphere expression differed between cell lines with PA-1 cells demonstrating strong sphere formation (Figure 2(C1)), while Caov-3 cells only formed small structures (Figure 2(C2)). After MSI dual knockdown, the average size of spheres was significantly reduced in PA-1 (*p* = 0.02) and Caov-3 (*p* = 0.004, Figure 2(D1)). The number of individual cellular structures was also reduced in PA-1 (*p* = 0.01) but not in Caov-3 (*p* = 0.9, Figure 2(D2)).

**Figure 3:**
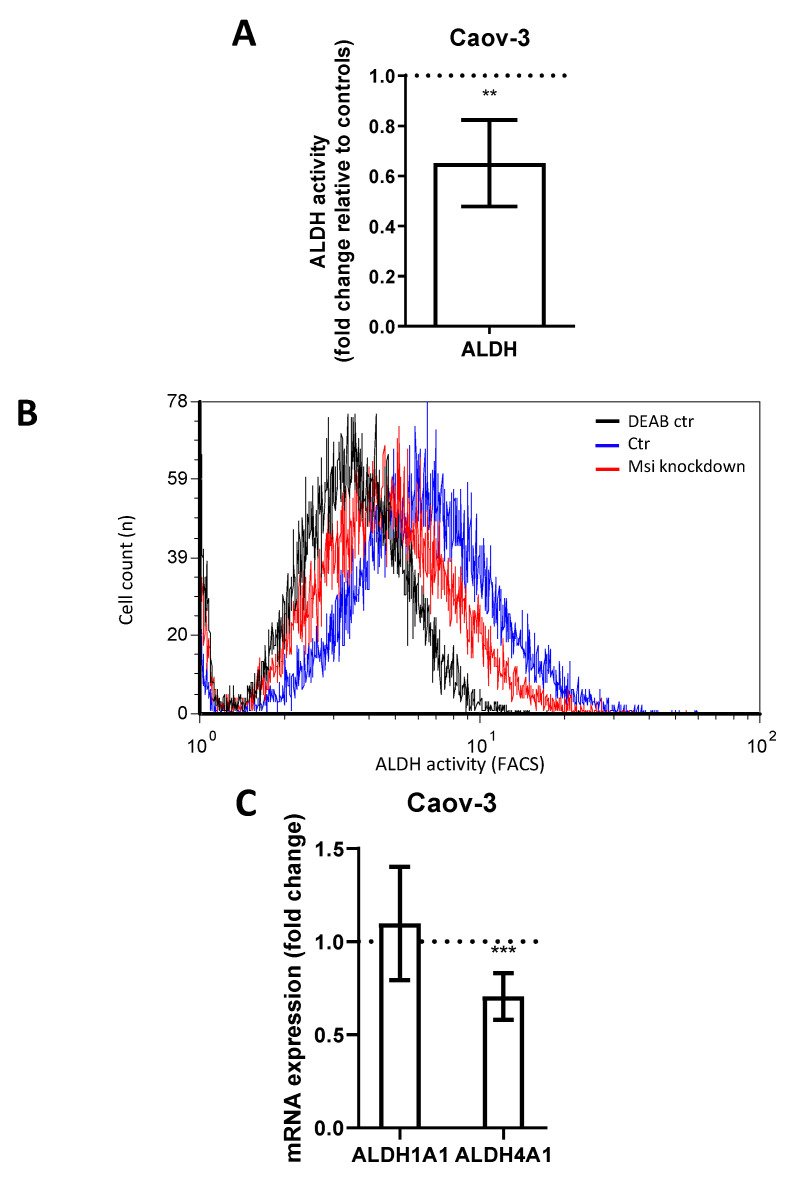
Aldehyde dehydrogenase activity in Caov-3 cells is downregulated after Musashi-1 and Musashi-2 inhibition. (**A**): Aldehyde dehydrogenase activity as determined by flow cytometry is reduced after dual inhibition compared to controls. (**B**): Representative flow cytometric measurements with “negative controls” shown in the presence of the ALDH-inhibitor diethylaminobenzaldehyde (*DEAB*). (**C**): ALDH4A1 is downregulated subsequent to dual inhibition, while ALDH1A1 is unchanged in quantitative polymerase chain reaction (qPCR) measurements. All experiments were repeated at least three times in triplets. *p* values < 0.05 were deemed significant (** *p* < 0.01; *** *p* < 0.001; error bars indicate standard error of the mean (s.e.m.)).

Aldehyde dehydrogenase (ALDH) is another CSC marker involved in the promotion of gene transcription and associated with cell differentiation and proliferation [23,24]. ALDH has previously been linked to MSI-1 expression [15]. In flow cytometry measurements, MSI-depleted Caov-3 cells demonstrated a loss of ALDH activity of 35% compared to controls (Figure 3A,B, *p* = 0.006). Subsequent qPCR analyses revealed that the subtype ALDH4A1 was significantly downregulated by 30% (Figure 3C, *p* = 0.01). Meanwhile, ALDH1A1 was expressed at relatively low levels (ct = 33.8), and expression did not change after MSI knockdown. Due to the low activity of ALDH in PA-1 (activity was not significantly different from diethylaminobenzaldehyde (*DEAB*) negative control, Supplementary Figure S5), the alterations could not be quantified in this cell line.

### 2.3. Cell Cycle Progression Is Modulated by MSI-1/-2 Knockdown

**Figure 4:**
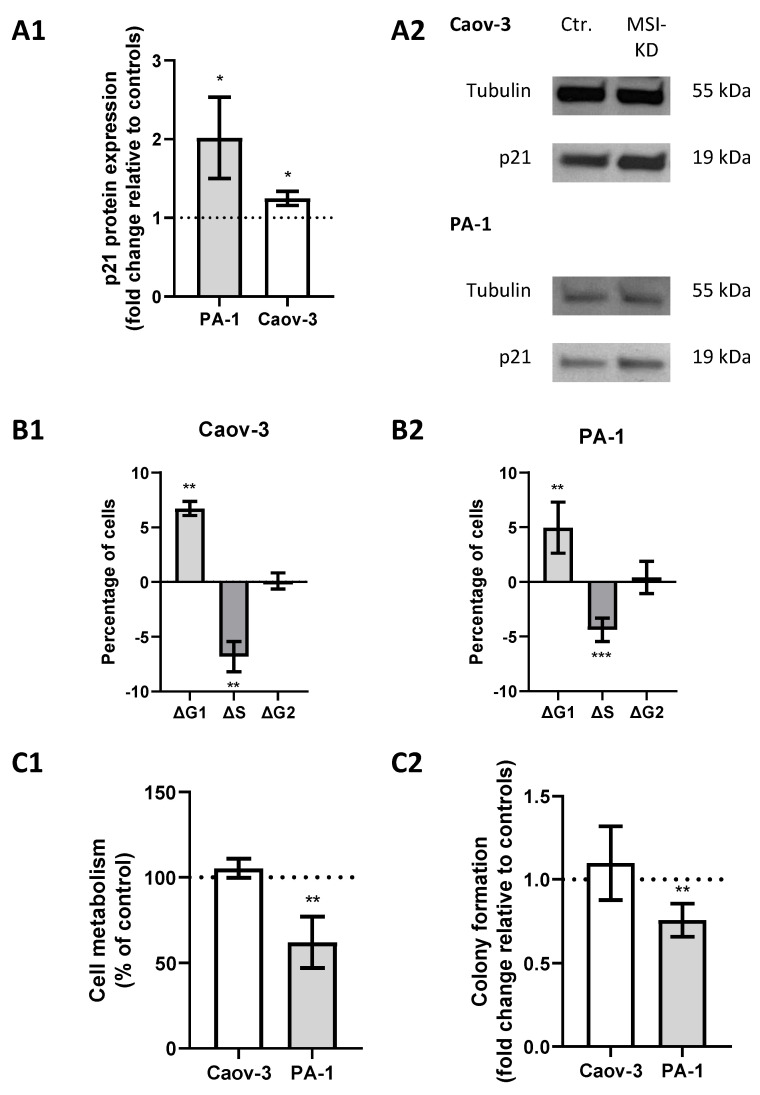
Musashi-mediated changes regarding cell cycle regulation. (**A1**): p21 is upregulated after Musashi dual inhibition in both cell lines with representative images in panel (**A2**). (**B1**,**B2**): Cells are more likely to be in the G1 phase and less likely to be in the S phase after Musashi dual inhibition in both cell lines. (**C1**,**C2**): Cell metabolism and colony formation are decreased in PA-1 cells, while no changes are seen in Caov-3 cells after Musashi dual inhibition. All experiments were repeated at least three times in duplicates. *p* values < 0.05 were deemed significant (* *p* < 0.05; ** *p* < 0.01; error bars indicate standard error of the mean (s.e.m.)).

A previous study showed direct regulation of p21 by MSI-1 and regulatory effects via NOTCH in endometrial carcinoma [25]. Since p21 is also closely linked to radioresistance via cell cycle modulation, we investigated its role in ovarian cancer. Western blot analyses confirmed an increase in p21 levels subsequent to MSI dual knockdown, since protein expression was increased 1.2-fold in Caov-3 (*p* = 0.03) and increased 2-fold in PA-1 cells (*p* = 0.03, Figure 4(A1,A2)).

Meanwhile, cell cycle analysis by flow cytometry illustrated a significant shift from S to G1 phase after MSI knockdown. Measurements indicated a significantly decreased proportion of cells in the S phase (*p* = 0.04 for Caov-3 and *p* = 0.025 for PA-1, respectively) and an increased proportion in the G1 phase (*p* = 0.002 and 0.008) after MSI knockdown in both cell lines (Figure 4(B1,B2)).

### 2.4. Cell Metabolic Activity Is Compromised Subsequent to MSI Dual Knockdown

Given the previously determined effects on CSCs and cell cycle progression, we hypothesized that MSI dual silencing may limit cell metabolism.

MTT assay confirmed that metabolic activity was reduced by 40% (*p* = 0.02, Figure 4(C1)) in MSI-depleted PA-1 cells. Similar effects could be seen in colony formation assays where MSI-deficient cells were 25% less likely to form colonies compared to controls (*p* = 0.005, Figure 4(C2)). Meanwhile, no changes were seen in Caov-3 cells.

### 2.5. Effects on Chemo- and Radiosensitivity

After quantifying a loss of metabolic activity in PA-1 cells after dual knockdown, we aimed to investigate whether knockdown cells showed additional increased sensitivity to therapy. Thus, an MTT assay was performed to test for chemosensitivity, and colony formation assays were used to detect effects on radiation response.

**Figure 5:**
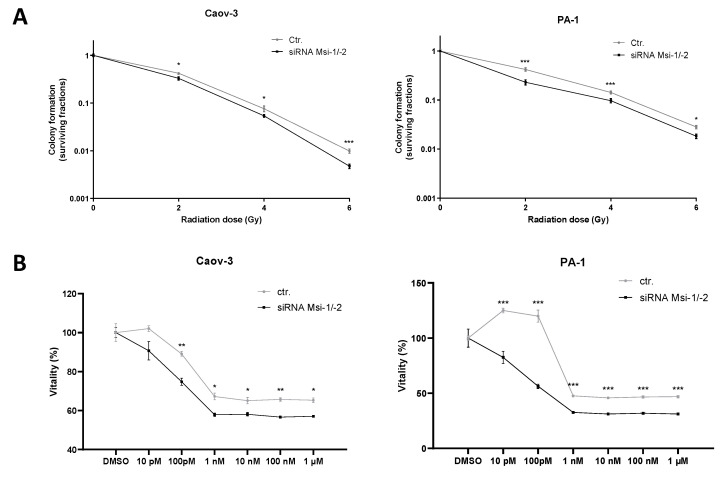
Musashi dual inhibition entails sensitization of ovarian cancer cells to radiation and chemotherapy. (**A**): Musashi knockdown Caov-3 and PA-1 cells consistently demonstrate reduced radioresistance after 2, 4, and 6 Gy of irradiation. (**B**): Caov-3 and PA-1 cells consistently demonstrate reduced chemoresistance to different paclitaxel doses. All experiments were repeated at least three times in duplicates. *p* values < 0.05 were deemed significant (* *p* < 0.05; ** *p* < 0.01; *** *p* < 0.001; error bars indicate standard error of the mean (s.e.m.)).

Colony formation assays showed an increased radiosensitivity for both cell lines after MSI knockdown as cells lost clonogenic ability after all tested doses of irradiation (*p* < 0.05 for all doses, Figure 5A, numerical results in Supplementary Tables S6 and S7). In Caov-3 cells, the effect was strongest for the highest radiation dose (6 Gy, *p* < 0.001), whereas in PA-1, the effect was most pronounced after smaller fractions (2 Gy, 4 Gy, *p* < 0.001 for both doses).

MSI-1 and -2 were both individually identified to affect response to paclitaxel treatment in ovarian cancer cells before [16,17]. Here, a significantly increased paclitaxel sensitivity after MSI-1/-2 double knockdown could be confirmed for both cell lines (Figure 5B).

## 3. Discussion

Here, we discuss findings regarding the relationship between the Musashi family, putative cancer stem cells and subsequent consequences for the therapeutic potential of targeting Musashi in ovarian cancer.

### 3.1. Musashi Dual Knockdown as an Attractive Therapeutic Option to Target Cancer Stem Cells

Musashi inhibition has previously been described as a therapeutic option to target cancer stem cells, thus reducing therapy resistance [10,26,27]. However, the exact interplay between MSI-1 and MSI-2 is unknown. Most studies have focused on either of the RNA-binding proteins. A number of therapeutically desirable goals have been achieved by targeting and inhibiting one of the Musashi proteins. In ovarian cancer, MSI-1 has been linked to overall survival and chemoresistance, while MSI-2 has also independently been linked to chemoresistance this way [15,16,17].

However, biomechanistic investigations have detailed the difficulty of targeting and inhibiting MSI-1 and MSI-2 separately according to their close similarity [22]. In fact, even the RNA-binding segment is overwhelmingly identical, indicating similar binding targets: In gastric cancer, a study found both proteins to have a 70% overlap in targets [28]. This previous investigation noted that “either MSI-1 or MSI-2 are sufficient to act upon target transcripts” [28], leading the authors to suggest that dual inhibition is essential for full abrogation of MSI effects. Our initial database analyses for this study further supported this approach given the close correlation between MSI-1 and MSI-2 in primary ovarian cancer samples. All this informed our decision to perform dual knockdown to understand MSI-related effects in ovarian cancer.

### 3.2. MSI Proteins Correlate with Cancer Stem Cell Related Genes Both In Vitro and In Vivo

Database analyses demonstrated significant associations between MSI protein expression and several genes associated with cancer stem cell characteristics.

CSCs are a small subpopulation of tumor cells with self-renewing potential; they have been identified as important drivers of metastasis and therapy resistance [29]. The notch signaling pathway is a key driver of cancer stem cell maintenance and targeted therapies are currently under investigation [30]. In other tumor entities, MSI proteins have been shown to bind the NOTCH inhibitor NUMB, thus increasing notch activity [1]. Given that both MSI-1 and MSI-2 are negatively correlated with NUMB and positively correlated with NOTCH-3 in our analyses, this seems applicable to ovarian cancer as well. Our knockdown experiments further underline these findings, as qPCR and Western blot analyses demonstrate an upregulation of NUMB and a downregulation of NOTCH-1 and NOTCH-3 after MSI dual knockdown.

ALDH, in particular its isoform ALDH1A1, is known to be a CSC marker with antiapoptotic and proliferative effects [23]. One previous study described a correlation between ALDH1 and MSI-1 [15] in a small ovarian cancer patient group. Interestingly, we could not reproduce this in our database analysis with a larger patient cohort. In Caov-3 cells, there was only a relatively low and unchanged expression of ALDH1A1 in vitro, whereas we showed a relatively high expression of ALDH4A1. MSI dual inhibition led to a significant reduction in ALDH4A1 expression in Caov-3 cells. Little is known about the role of ALDH4A1 in cancer. High expressions of this isoform were seen in prostate cancer [31] and recently described in Hodgkin’s lymphoma [32] by our group. In ovarian cancer, ALDH4 has been shown to be protective against reactive oxygen species (ROS) and subsequent DNA damage [33]. This points to a radiation-relevant role for MSI proteins, as ROS-based DNA corruption is a key mechanism of radiation-induced cell damage.

The Aldeflour assay was originally designed to measure ALDH1 activity specifically, but there is evidence for a rather unspecific activity measurement of many ALDH isoforms [34]. Since our investigations showed a significantly reduced ALDH activity after MSI knockdown in Caov-3 cells, we predominantly attribute these effects to reduced expression of ALDH4A1.

In PA-1, ALDH activity was extremely low, precluding us from detailed analyses. This is in line with a previous study that found less than 2% of non-sorted PA-1 cells to be ALDH-positive [35].

Sphere forming ovarian cancer cells exhibit numerous CSC characteristics, including increased tumor formation, metastasis and therapy resistance [36]. They have been linked to numerous CSC genes, including ALDH [37] and NOTCH [38]. Sphere formation in breast [9] and lung [39] cancer was reduced after MSI knockdown. Given these potential mechanistic links and similar findings in other tumor entities, our results indicating loss of sphere formation fit well with previous knowledge. Interestingly, while the average size of spheres was downregulated in both cell lines, the number of structures (including non-spheres) was only reduced in PA-1, not in Caov-3 cells. This foreshadows findings from proliferation and cell metabolism assays and will be discussed below.

In sum, we found that MSI targeting results in decreased putative CSC characteristics. As CSCs are key to therapy resistance, including in ovarian cancer [40,41], our findings prompted us to perform subsequent experiments to assess the therapeutic relevance of MSI knockdown.

### 3.3. P21 Is Upregulated after Musashi Inhibition Resulting in Cell Cycle Arrest

P21 is known to be closely related to MSI and MSI-related pathways [9]. In our database analysis, p21 was negatively associated with MSI-2 and trended towards a negative association with MSI-1. In vitro, p21 was upregulated after MSI dual inhibition. Several possible mechanistic explanations come to mind.

In endothelial cells, NOTCH-1 functions as an inhibitor of p21 [42]. p21 was also previously described as a downstream target of NOTCH-1 in cancers, e.g., endometrial carcinoma and colorectal carcinoma [25,43]. Here, we established a reduction in notch pathway element expression after MSI knockdown. Thus, notch-associated p21 inhibition is likely lifted in this scenario and may explain increased p21 expression.

MYC, a transcription factor and CSC marker previously reported to be regulated by Musashi [26], is also known to repress p21 [44,45]. In our database analyses, we demonstrate that MSI-1 is positively associated with MYC, while MYC is down after MSI inhibition in PA-1 cells. Thus, reduced MYC expression after MSI inhibition may lead to an increase in p21 gene expression.

Lastly, MSI-1 itself is known to be a direct translational repressor of p21 [46].

Therefore, there are multiple intertwined MSI-related pathways that may explain the increased p21 protein expression after MSI knockdown.

The upregulation of p21 results in cell cycle shifts: We quantified a shift from S- to G1-phase in our study in both cell lines. Subsequent experiments confirmed decreased metabolic activity and baseline colony formation in PA-1, but not in Caov-3 cells. These findings underline the critical role MSI and p21 play in ovarian cancer progression. They also support the previously reported anti-proliferative effect associated with MSI-2 targeting, where increased apoptosis was seen [17].

Notably, Caov-3 cells behaved differently from PA-1 cells by not exhibiting a proliferation decrease or loss in cell metabolism after dual knockdown. As noted above, the number of living cell structures in 3D CSC spheroid culture was similarly unchanged compared to controls, again different from PA-1 cells. We believe that the key reason for these findings is a general difference between the teratoma cell line PA-1 and adenocarcinoma Caov-3 cells: First, studies have found PA-1 to show a much higher level of baseline proliferation compared to Caov-3 with median doubling times roughly 15 vs. 30 h [47]. Thus, the changes in cell cycle progression we saw in both cell lines may have had more dramatic ramifications in the fast-proliferating PA-1 cells compared to the more slowly growing Caov-3 line. Second, PA-1 has been described to be more vulnerable to therapeutic interventions than Caov-3, regarding radiation [47], TKIs (Crizotinib IC50 162 vs. 340 nM) [48], C-mediated toxicity [49], and use of all-trans retinoic acid (ATRA) [50]. Our differing Western blot results for the antiproliferative marker p21 with a strong increase in PA-1 and only modest elevation in Caov-3 provide additional evidence for this. Thus, our results and other studies suggest that PA-1 cells are more vulnerable to therapeutic interventions and dual knockdown alone is not sufficient to target more resistant Caov-3 cells.

### 3.4. MSI-1/-2 Expression Affects Radio- and Chemoresistance In Vitro

MSI downregulation resulted in significant radio- and chemosensitizing effects. While chemosensitization has been described for both MSI-1 knockdown and MSI-2 targeting independently in ovarian cancer cells [16,17], radiation effects have not previously been investigated. Our study demonstrates that dual knockdown results in a chemosensitization effect similar to single MSI knockdown and additionally radiosensitizes cells. In the case of PA-1 cells, both effects are additional to the anti-proliferative effect seen after knockdown alone. These findings may make dual MSI targeting a highly interesting option given the therapeutically favorable consequences. Interestingly, effects after both chemo- and radiotherapy were similar between cell lines PA-1 and Caov-3. While Caov-3 cells did not show a proliferation decrease after dual knockdown alone, we hypothesize that dual knockdown and chemo-/radiotherapy may jointly overcome the more pronounced resistance mechanisms in Caov-3 cells.

Again, multiple mechanistic explanations may help explain the findings.

- The notch pathway has already been described as an important factor of radio- and chemoresistance in several entities [51,52,53,54]. The role of MYC is similar [55,56,57]. Both NOTCH and MYC—which are downregulated by MSI knockdown—are important for cancer stem cell maintenance. CSCs have been identified to be associated with increased radioresistance [58].
- As discussed above, ALDH4 has been linked to increased resistance to oxidative stress [33]. Given the downregulation of ALDH4 after MSI knockdown, cells may become more susceptible to radiation-induced, ROS-mediated DNA damage.
- Low levels of p21 are also associated with higher radioresistance in lung cancer [59]. It is long known that radiation response changes through the different cell cycle phases from being most resistant through the S-phase to being more sensitive in G1-phase and most sensitive in the G2/M-phase [60]. Thus, we also explain increased radiosensitivity by the previously described p21-mediated cell cycle arrest with more cells accumulating in a more sensitive cell cycle phase.

There are some limitations to this study. First, even though correlations between MSI-1/-2 and the investigated genes were based on patient probes, research on the mechanistically involved pathways was only performed in vitro. Findings are in line with previous data in other cancer entities, but the in vivo applicability of the postulated effects needs to be validated in further research. Second, our use of two cell lines with two different ovarian cancer subtypes, adenocarcinoma and teratoma, demonstrated some differences in results. While most results show a broad applicability of our findings, pre-therapeutic cell metabolism and colony formation was only impaired in PA-1 (teratoma) after MSI knockdown, while ALDH was only relevantly expressed in Caov-3 (adenocarcinoma). This suggests that a more detailed look at the subtypes might be necessary to identify some specific differences. However, therapeutically relevant findings did not differ. Third, chemoresistance measurements indicated an unchanged response to therapy despite increasing paclitaxel concentrations between 1 nM and 1 μM. While unlikely and surprising, this is consistent with a previous study [61]. Independent of these findings, chemosensitization effects of MSI dual knockdown were consistent across all chemotherapy doses tested, underlining the validity of our conclusions. Fourth, not all qPCR analyses could be validated by Western blots. Western blots of MSI-2 were difficult to perform due to cross-reactions, especially between MSI-1 and MSI-2 (molecular weight of 39 kDa and 35 kDa, respectively), probably given their similarity. However, Western blot was successful for most targets, especially those pertinent to key findings. Western blots largely confirmed qPCR findings.

Taken together, our study demonstrates relevant associations between Musashi RNA-binding proteins and key cancer stem cell and cell cycle genes in a large ovarian cancer patient dataset. It subsequently shows that Musashi-1 and Musashi-2 dual inhibition results in numerous favorable changes in ovarian cancer cells in vitro: a downregulation of important cancer stem cell-associated genes, especially NOTCH-1, NOTCH-3 and ALDH4A1; a decrease in sphere formation; cell cycle arrest via p21; and a loss in metabolic activity. Finally, MSI dual knockdown leads to decreased radio- and chemoresistance. We conclude that targeting both MSI proteins is a promising venue to impede ovarian cancer growth and address therapy resistance.

## 4. Materials and Methods

### 4.1. Cell Lines, Cultivation and Transfection

The ovarian cancer cell lines Caov-3 (adenocarcinoma) and PA-1 (ovarian teratocarcinoma) were obtained from American Type Culture Collection (ATCC)/LGC Standards (Wesel, Germany). Cells were cultured in the recommended media, DMEM for Caov-3 and MEM-Eagle for PA-1 (both PAN Biotech, Aidenbach, Germany) at 37 °C in a H_2_O saturated atmosphere containing 5% CO_2_. The media were supplemented with 10% Fetal calf serum (FCS; PAN Biotech, Aidenbach, Germany), 1% Pen/Strep (PAN Biotech, Aidenbach, Germany), and 25 mM Hepes (Roth, Karlsruhe, Germany).

MSI double knockdown was performed via transient siRNA transfection with 10 nM of both MSI-1 and MSI-2 siRNA (Supplementary Table S1). The transient transfection (stable for at least 72 h) was conducted using Lipofectamine RNAiMAX (ThermoFisher Scientific, Waltham, MA, USA) according to the manufacturer’s instructions. Validation of successful knockdown was performed via quantitative polymerase chain reaction (qPCR) analysis (Supplementary Figure S1) and Western Blot analysis (Supplementary Figure S2).

### 4.2. RNA Isolation, Reverse Transcription and qPCR

For qPCR, RNA was isolated 72 h after transfection with the RNeasy^®^ Mini Kit (QIAGEN, Venlo, The Netherlands). The cDNA Reverse Transcription Kit (BD Pharmingen, Franklin Lakes, NJ, USA) was used for reverse transcription. Isolation and transcription kits were utilized according to the manufacturer’s protocol.

qPCR was conducted either with a TaqMan^®^ Gene Expression Assay (Thermo Fisher Scientific, Waltham, MA, USA) for MSI-1, MSI-2 and NUMB, normalizing to 18S-rRNA-Expression, or with SYTO9 by using specific primers (for NOTCH-1, NOTCH-2, NOTCH-3), normalizing to HPRT expression. The results were expressed as fold change and analyzed using the 2^−∆∆ct^ method. TaqMan probes were obtained from Applied Biosystems (Foster City, CA, USA) while primers were from QIAGEN (Venlo, Netherlands) and Biolegio (Nijmegen, The Netherlands). Detailed information about probes and primers can be found in Supplementary Tables S2 and S3.

### 4.3. Western Blot

For Western blot analyses, proteins were isolated 72 h after transfection as previously described [62]. For electrophoresis, a 4% to 20% precast gradient gel (Bio-Rad Laboratories, Feldkirchen, Germany) was loaded with 30 µg of whole protein extract and 15 µg of a prestained protein standard (ThermoFisher Scientific, Waltham, MA, USA) for molecular weight evaluation. Electrophoresis, protein transfer, and antibody staining were performed as previously described [10]. Incubation with the primary antibody was performed overnight at 4 °C and with the secondary antibody for two hours at room temperature. Antibody details are specified in Supplementary Table S4. Chemiluminescence of bound antibodies was induced using ECL substrate (ThermoFisher Scientific, Waltham, MA, USA) and visualized on X-ray films. Afterwards, the membrane was stripped and then stained again to visualize another protein of interest. The intensity of specific bands was measured with ImageJ Ver. 1.53 (NIH, Bethesda, MD, USA) and normalized to tubulin as the reference protein. Importantly, samples and respective controls were stained on the same gel.

### 4.4. ALDH Quantification

An Aldefluor^TM^ Kit (Stem Cell Technologies, Vancouver, BC, Canada) was used to visualize cellular ALDH activity. Cells were stained according to the manufacturer’s instructions 48 h after transfection. Quantification of ALDH activity was measured by flow cytometry (CyFlow Space, Sysmex/Partec, Görlitz, Germany). A 488 nm argon laser was used for excitation and fluorescence emission was measured at 527 nm in FL1. A region gate was set in histograms and the mean fluorescence intensity (MFI) was calculated to quantify fluorescence intensity using FloMax software (Quantum Analysis, Münster, Germany).

### 4.5. Cell Cycle Analysis

Cell cycle analysis was performed 48 h after transfection using 4′,6-diamidino-2phenylindole (DAPI; CyStain, Sysmex/Partec, Münster, Germany), as previously described [10]. FCS Express (Pasadena, CA, USA) multicycle analysis was used to determine the cell cycle distribution.

### 4.6. Cell Metabolism and Chemosensitivity Assay

MTT assay was used to determine metabolic activity and chemosensitivity in control cells and after dual inhibition as previously described [63]. Paclitaxel was therefore used as chemotherapeutic agent with titrated concentrations (10 pm–1 µm). Cells were incubated for 96 h with DMSO containing the determined concentration of Paclitaxel. Then MTT assay was performed with a TriStar LB942 ELISA-Reader (Berthold Technologies, Bad Wildbad, Germany) at 570 nm. The measurements were compared to controls treated with DMSO only.

### 4.7. Colony Formation Assay

Cells were irradiated on a TrueBeam linear accelerator (Varian, Palo Alto, CA, USA) 24 h after transfection, with doses of 2, 4, and 6 Gy using a dose rate of 4.8 Gy per minute, as previously described [64]. Immediately after irradiation, cells were detached and counted, and a predefined number of cells were seeded into petri dishes (Thermo Fisher Scientific, Waltham, MA, USA). After 14 days of cultivation, colonies—defined as a group of more than 40 cells—were counted. Results were obtained by calculating the surviving fraction (%SF). %SF was defined as %SF = PE (irradiated cells)/PE (control) with the plating efficiency (PE) being defined as PE = number of colonies/number of seeded cells.

### 4.8. Sphere Formation Assay

A predefined number of cells was seeded in suspension culture plates (Greiner Bio-One, Kremsmünster, Austria) 24 h after transfection. Cells were then cultivated with Spheromax CSC Medium (Promocell, Heidelberg, Germany). After 7 days, spheres were counted (number per microscopic field of view, 50× magnification) and the area was measured using an open source ImageJ macro, manually correcting visually incorrect counts [65].

### 4.9. Database Analysis

Using open source data from the GSE74357 dataset, including gene expression analyses from 529 ovarian cancer samples [66], the correlation between mRNA expression levels of MSI-1/-2 and pre-defined genes of interest was analyzed. Genes and their aligned feature number can be found in Supplementary Table S5.

### 4.10. Statistics

All in vitro experiments were performed independently three times in duplicates. Significances were tested using Student’s *t*-test. The threshold of significance was defined as *p* = 0.05. Correlation of mRNA expression levels in the database was analyzed with Spearman’s rank correlation, defining level of significance as *p* < 0.05.

Statistical analyses and graphs were generated with Prism 8 (GraphPad Software, San Diego, CA, USA). Flow cytometry data was visualized using FCS Express 7 (Pasadena, CA, USA) or FloMax (Quantum Analysis, Münster, Germany).

## Data Availability

All supporting data can be found in the manuscript.

## Supplementary Materials

The following are available online at https://www.mdpi.com/article/10.3390/ijms222111502/s1.
