# Supplementary material for: Dual Knockdown of Musashi RNA-Binding Proteins MSI-1 and MSI-2 Attenuates Putative Cancer Stem Cell Characteristics and Therapy Resistance in Ovarian Cancer Cells
Source: Int J Mol Sci. 2021 Oct 25;22(21):11502. doi: 10.3390/ijms222111502 (PMC8584030; doi:10.3390/ijms222111502)

Supplementary Material

Supplementary Table S1. SiRNAs used for knockdown.

siRNA	Manufacturer	Manufacturer's ID	UniGene ID
MSI-1	ThermoFisher Scientific	S8980	Hs.158311
MSI-2	ThermoFisher Scientific	S42757	Hs.658922
Negative Control	ThermoFisher Scientific	4390843	-

Supplementary Table S2. TaqMan probes used for qPCR.

Gene	Manufacturer	Primer sequence
NUMB	ThermoFisher Scientific Hs00377772_m1	
18S	ThermoFisher Scientific Hs99999901_s1	
MSI-1	ThermoFisher Scientific Hs00159291_m1	*Full product details can be found on the manufacturer's website using the catalogue number*
MSI-2	ThermoFisher Scientific Hs00292670_m1	
ALDH1A1	ThermoFisher Scientific Hs00167445_m1	
ALDH4A1	ThermoFisher Scientific Hs00186689_m1	

Supplementary Table S3. Primers used for qPCR with SYTO9.

Gene	Manufacturer	Primer sequence
HPRT	Qiagen *(QT00059066)*	*Full product details can be found on the manufacturer's website using the catalogue number.*
NOTCH-1	Biolegio	Fwd: GGTGAGACCTGCCTGAATG<br>Rev: GTTGGGGTCCTGGCATC
NOTCH-2	Biolegio	Fwd: AACTGTCAGACCCTGGTGAAC<br>Rev: CGACAAGTGTAGCCTCCAATC
NOTCH-3	Biolegio	Fwd: TGTGCAAATGGAGGTCGTT<br>Rev: CCTGAGTGACAGGGGTCCT

Supplementary Table S4. Antibodies used for Western Blotting.

Target	Manufacturer	Catalogue number
NUMB	Santa Cruz	sc-136554
NOTCH-1	Santa Cruz	sc-376403
NOTCH-3	Cell Signaling Technology	5276
p21	Cell Signaling Technology	2947
MSI-1	R & D Systems	AF2628
MSI-2	Santa Cruz	sc-517212

Tubulin	Santa Cruz	sc-5286
Anti-Rabbit IgG HRP	R & D Systems	HAF008
Anti-Mouse IgG HRP	R & D Systems	HAF007

Supplementary Table S5. Genes analyzed in the ovarian cancer database.

Gene	ID
MSI-1	A_23_P139795
MSI-2	A_23_P369479
NUMB	A_23_P88381
NOTCH-3	A_24_P399606
CDKN1A (p21)	A_23_P59210
MYC	A_24_P178011
ALDH4A1	A_24_P263036

Supplementary Table S6: Surviving fractions after irradiation in PA-1 cells with and without double knockdown.

Radiation dose	Control	MSI-1/-2 Knockdown
**0 Gy**	100	100
**2 Gy**	41,94	22,98
**4 Gy**	14,24	9,68
**6 Gy**	2,82	1,83

Supplementary Table S7: Surviving fractions after irradiation in Caov-3 cells with and without double knockdown.

Radiation dose	Control	MSI-1/-2 Knockdown
**0 Gy**	100	100
**2 Gy**	42,03	32,92
**4 Gy**	7,18	5,82
**6 Gy**	0,99	0,47

*Supplementary Figure S1. Musashi-1 (MSI-1) and Musashi-2 (MSI-2) RNA levels in both cell lines after knockdown with MSI-1 and MSI-2 siRNA, quantified by quantitative polymerase chain reaction (qPCR). mRNAs were repressed by 74% to 85% percent. Cells were transfected with a control siRNA and MSI-1 / -2 siRNAs as detailed in the methods section (at least n = 3, *** p < 0.001, error bars indicate standard error of the mean (s.e.m.)).*

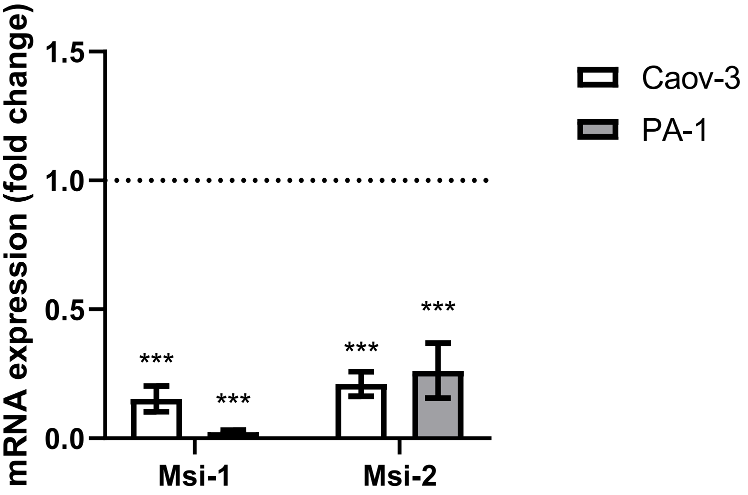

*Supplementary figure S2. Musashi-1 (MSI-1) and Musashi-2 (MSI-2) protein levels in both cell lines after knockdown with MSI-1 and MSI-2 siRNA, quantified by Western Blot analysis. Proteins were repressed by at least 50% – 90%, except for MSI-2 in Caov-3. Cells were transfected with a control siRNA and MSI-1/-2 siRNAs as detailed in the methods section (at least n = 3, *** p < 0.001, error bars indicate standard error of the mean (s.e.m.))* ***A****: Western Blot results.* ***B****: representative Western Blot staining.*

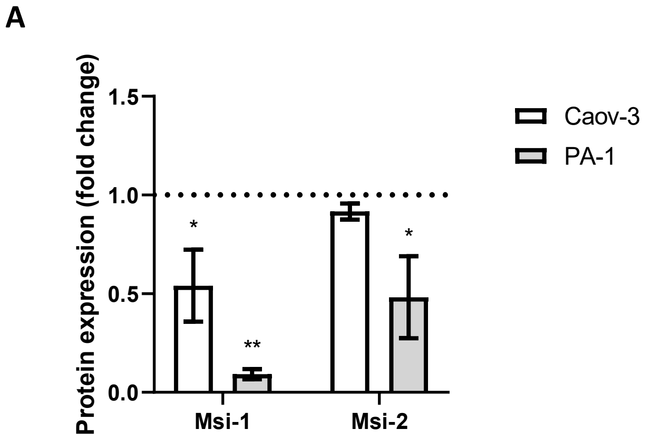

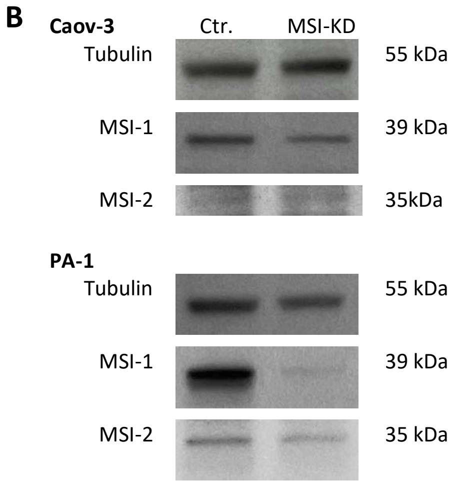

Supplementary figure S3. Relative protein expression levels in Caov-3 cells. The expression was normalized to Tubulin as reference protein (error bars indicate standard error of the mean (s.e.m.)).

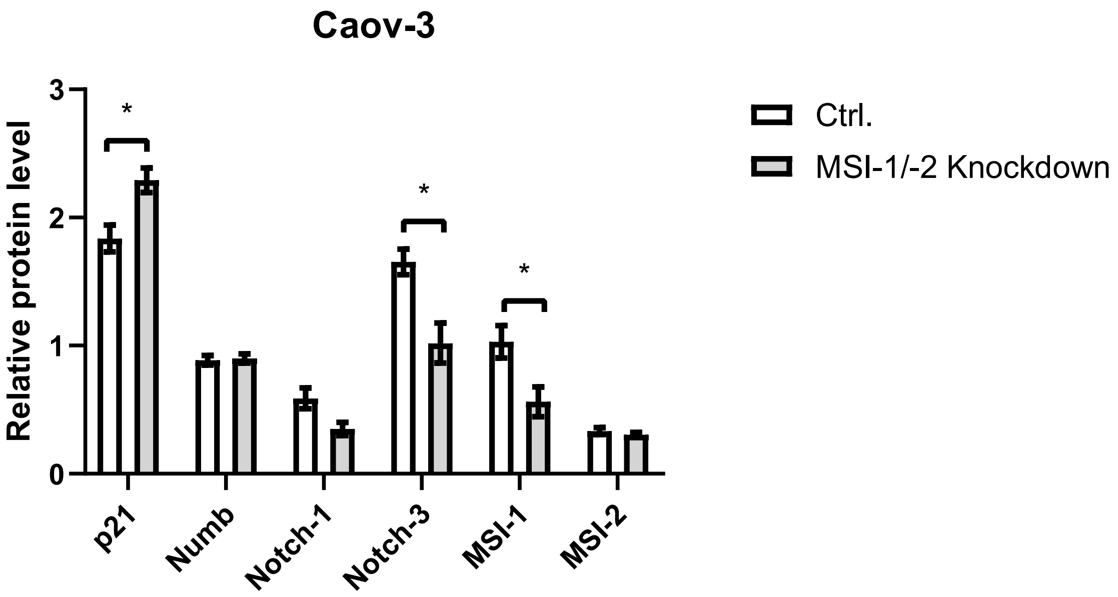

Supplementary figure S4. Relative protein expression levels in PA-1 cells. The expression was normalized to Tubulin as reference protein (error bars indicate standard error of the mean (s.e.m.)).

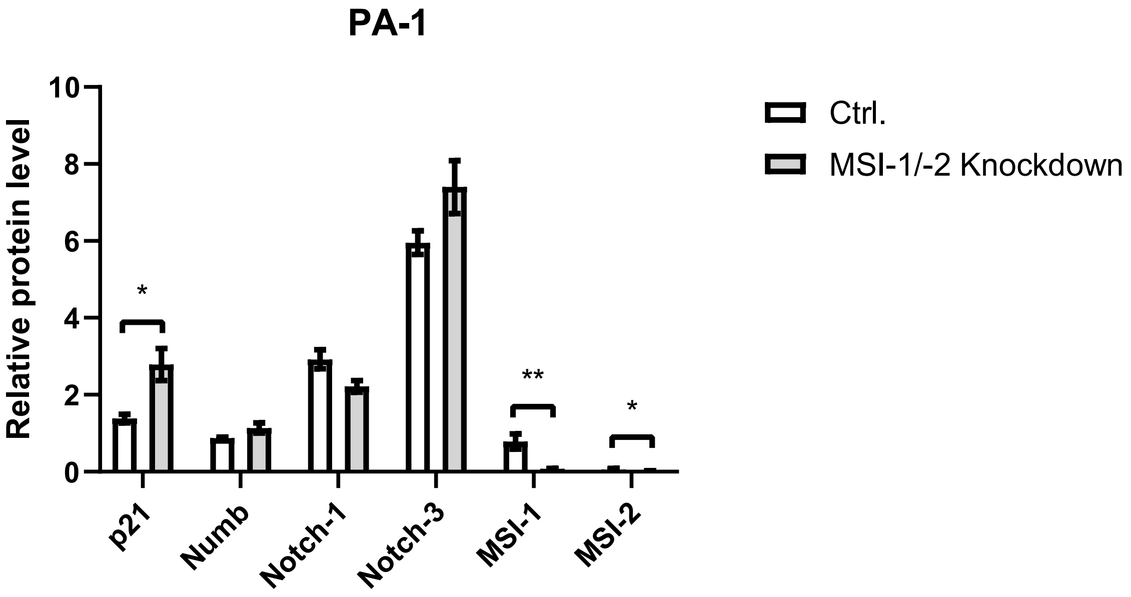

Supplementary Figure S5. Representative flow cytometric measurement of ALDH activity using Aldeflour assay in PA-1 cells, shown with a logarithmic x scale. ALDH activity barely exceeded the diethylaminobenzaldehyde (DEAB) control on baseline and no significant changes after knockdown were found.

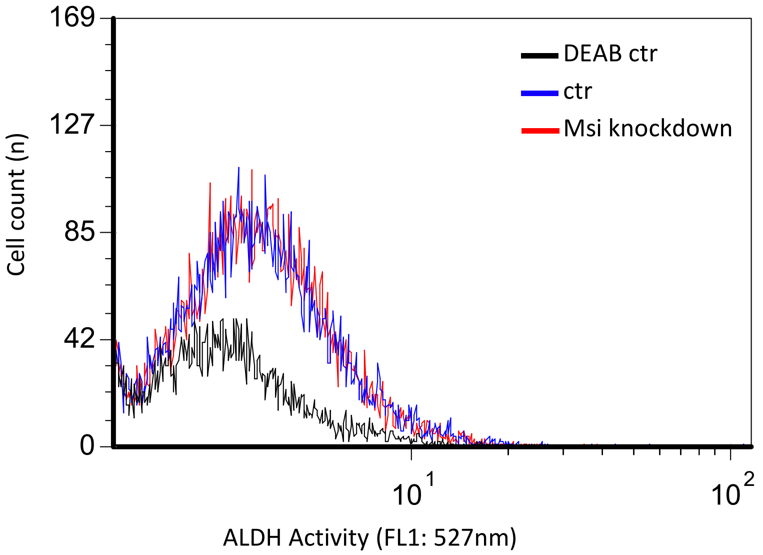

Supplement: Supplementary file 1 [file ijms-22-11502-s001.zip › ijms-1396757-supplementary.pdf]
